# LEAP2 Regulates Insulin Secretion from INS-1E Beta Cells and Rat Pancreatic Islets: An In Vitro Study

**DOI:** 10.3390/ijms27177536

**Published:** 2026-08-23

**Authors:** Oskar Sosiński, Ewa Pruszyńska-Oszmałek, Maciej Sassek, Natalia Leciejewska, Piotr Pawlak, Paweł Antoni Kołodziejski

**Affiliations:** 1 Department of Animal Physiology and Biochemistry, Poznan University of Life Sciences, Wolynska 35, 60-637 Poznan, Poland; oskar.sosinski@up.poznan.pl (O.S.); maciej.sassek@up.poznan.pl (M.S.); natalia.leciejewska@up.poznan.pl (N.L.); 2 Department of Genetics and Animal Breeding, Poznan University of Life Sciences, Wolynska 33, 60-637 Poznan, Poland; piotr.pawlak@up.poznan.pl

**Keywords:** LEAP2, GHSR-1a, ghrelin receptor, insulin secretion, INS-1E cells, pancreatic islets

## Abstract

LEAP2 is an endogenous antagonist and inverse agonist of GHSR-1a, the receptor for acyl-ghrelin. Although LEAP2 is increasingly recognized as a regulator of appetite and glucose homeostasis, its direct effects on pancreatic endocrine cells remain incompletely understood. The present in vitro study evaluated whether LEAP2 modulates insulin secretion and the expression of insulin-related genes in INS-1E beta cells and isolated rat pancreatic islets. LEAP2 treatment altered selected components of the insulin-related expression profile in a model- and time-dependent manner. In INS-1E cells, LEAP2 increased insulin protein expression after 6 h and IRSII protein expression after 24 h, whereas effects on mRNA expression were variable. In isolated rat islets, LEAP2 increased *INS2* mRNA expression and decreased *GHSR-1a* and preproghrelin mRNA expression under the tested conditions. Acute exposure to 100 nM LEAP2 increased basal insulin release from both INS-1E cells and isolated islets. Immunofluorescence and Western blot analyses detected LEAP2- and GHSR-1a-related immunoreactivity in pancreatic models. LEAP2 did not adversely affect INS-1E cell metabolic activity, proliferation, or apoptosis. Overall, these data suggest that LEAP2 can modulate pancreatic endocrine cell function under in vitro conditions, but further studies using standard glucose-stimulated insulin secretion protocols and in vivo models are required to define its physiological relevance.

## 1. Introduction

Liver-Enriched Antimicrobial Peptide 2 (LEAP2) is a 40-amino-acid peptide first identified in human blood ultrafiltrate in 2003 [1]. It is produced predominantly in the liver and gastrointestinal tract, although lower expression has also been reported in other tissues, including metabolically active organs. Although LEAP2 was initially described as an antimicrobial peptide, circulating concentrations are considered too low to account for a major antimicrobial effect under physiological conditions [2]. Instead, growing evidence indicates that LEAP2 plays a key role in metabolic regulation. In particular, LEAP2 has been identified as an endogenous antagonist of the growth hormone secretagogue receptor (GHSR-1a), the receptor for ghrelin, a hormone that stimulates appetite and regulates energy homeostasis [1,2,3]. LEAP2 has been shown to inhibit ghrelin-induced signaling and, under certain conditions, to act as an inverse agonist of GHSR-1a, thereby suppressing its constitutive activity [4,5,6].

The LEAP2-GHSR axis has attracted increasing attention in the context of glucose metabolism, insulin secretion and metabolic disease [7]. Ghrelin has been reported to exert insulinostatic effects in several experimental systems, whereas LEAP2 may counteract ghrelin-dependent signaling [2]. However, the direct contribution of LEAP2 to pancreatic beta-cell function remains insufficiently defined [8]. This is particularly relevant because changes in LEAP2 concentration have been linked with obesity, diabetes and altered postprandial endocrine responses [9], but it remains unclear whether these associations reflect direct pancreatic actions or indirect systemic effects.

Pancreatic islets are heterogeneous endocrine micro-organs composed mainly of insulin-producing beta cells, glucagon-producing alpha cells and somatostatin-producing delta cells [10,11]. Therefore, observations obtained in isolated islets may reflect direct beta-cell effects as well as indirect paracrine interactions between different endocrine cell types. In contrast, INS-1E cells provide a controlled and widely used beta-cell line model, but they do not fully reproduce the phenotype, architecture and regulatory environment of primary beta cells in vivo [12]. Consequently, the use of both models may provide complementary information, provided that their limitations are clearly acknowledged.

In this study, we investigated the effects of LEAP2 on insulin expression and basal insulin release in vitro using INS-1E cells and isolated rat pancreatic islets. We assessed selected changes at mRNA and protein levels and examined whether LEAP2 affected INS-1E cell metabolic activity, proliferation and apoptosis. In addition, we evaluated LEAP2- and GHSR-1a-related immunoreactivity in INS-1E cells, isolated islets and rat pancreatic tissue. The study was designed as an exploratory in vitro investigation and does not by itself establish the physiological effects of endogenous LEAP2 in vivo.

## 2. Results

### 2.1. Localization of LEAP2 and GHSR-1a

**Figure 1:**
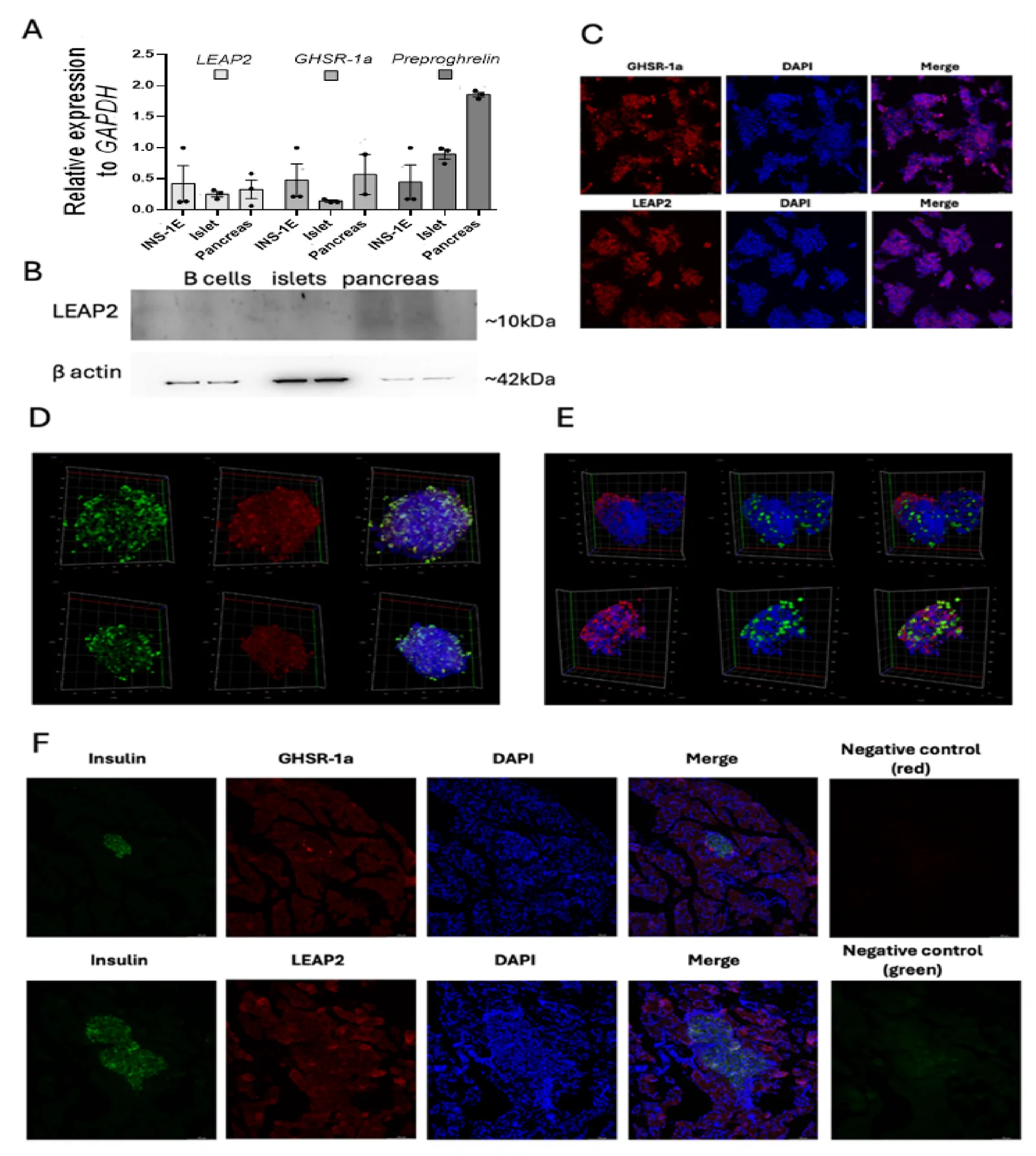
LEAP2, GHSR-1a and preproghrelin mRNA expression in INS-1E cell line, isolated rat pancreatic islets and rat pancreas, *n* = 3 (**A**). LEAP2 protein expression by Western blotting (**B**). Immunofluorescence staining: for LEAP2 and GHSR-1a proteins in INS-1e cell line (**C**), for LEAP2 (red) and insulin (green) in rat islet (**D**), for GHSR-1a (red) and insulin (green) in rat islet (**E**) and for LEAP2 and GHSR-1a proteins in pancreas (**F**).

Real-time PCR detected *LEAP2*, *GHSR-1a* and preproghrelin transcripts in INS-1E cells, isolated rat pancreatic islets and pancreatic tissue (Figure 1A). Western blotting detected LEAP2-related immunoreactive bands in the examined samples (Figure 1B). Immunofluorescence staining further showed LEAP2- and GHSR-1a-related immunoreactivity in INS-1E cells, isolated islets and pancreatic tissue (Figure 1C–F). Figure 1F also includes negative control for pancreas slides incubated with a secondary antibody only, to prove that the obtained results are not an effect of autofluorescence. Because LEAP2 signal intensity was low and antibody specificity was not confirmed by knockout tissue or peptide competition, these localization data should be interpreted cautiously.

### 2.2. LEAP2 Modulates Expression of Insulin and Related Genes in β Cells and Islets

**Figure 2:**
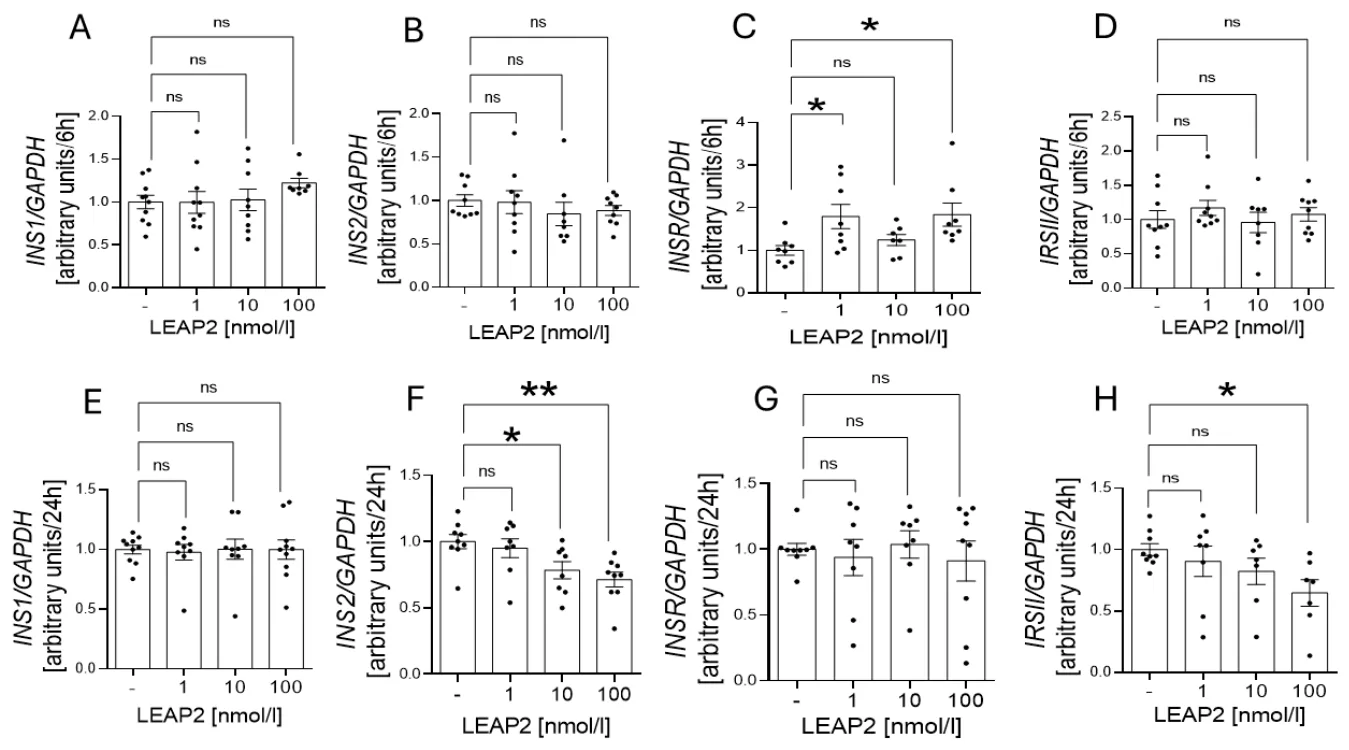
Influence of LEAP2 on mRNA expression of INS1 (**A**,**E**) and INS2 (**B**,**F**), INSR (**C**,**G**) and IRSII (**D**,**H**) in INS-1E cells after for 6 h and for 24 h. Results are presented as mean ± SEM. Statistically significant differences between the means of the studied groups are indicated as follows: ns = not significant; * *p* < 0.05; ** *p* < 0.01, experiment was performed thrice using *n* = 3 per each.

Real-time PCR for INS-1E cells showed that after 6 h, *INSR* mRNA expression was upregulated (*p* < 0.05) when exposed to 1 and 100 nM LEAP2 (Figure 2C), while insulin 1 (Figure 2A), insulin 2 (Figure 2B), and *IRS*II (Figure 2D) showed no changes.

After 24 h, lower insulin 2 expression was seen at 10 nM (*p* < 0.05) and at 100 nM (*p* < 0.01) LEAP2 concentrations (Figure 2F). *IRS*II showed lower mRNA expression (*p* < 0.05) when exposed to 100 nM concentration (Figure 2H). Insulin 1 (Figure 2E) and *INSR* (Figure 2G) showed no changes.

**Figure 3:**
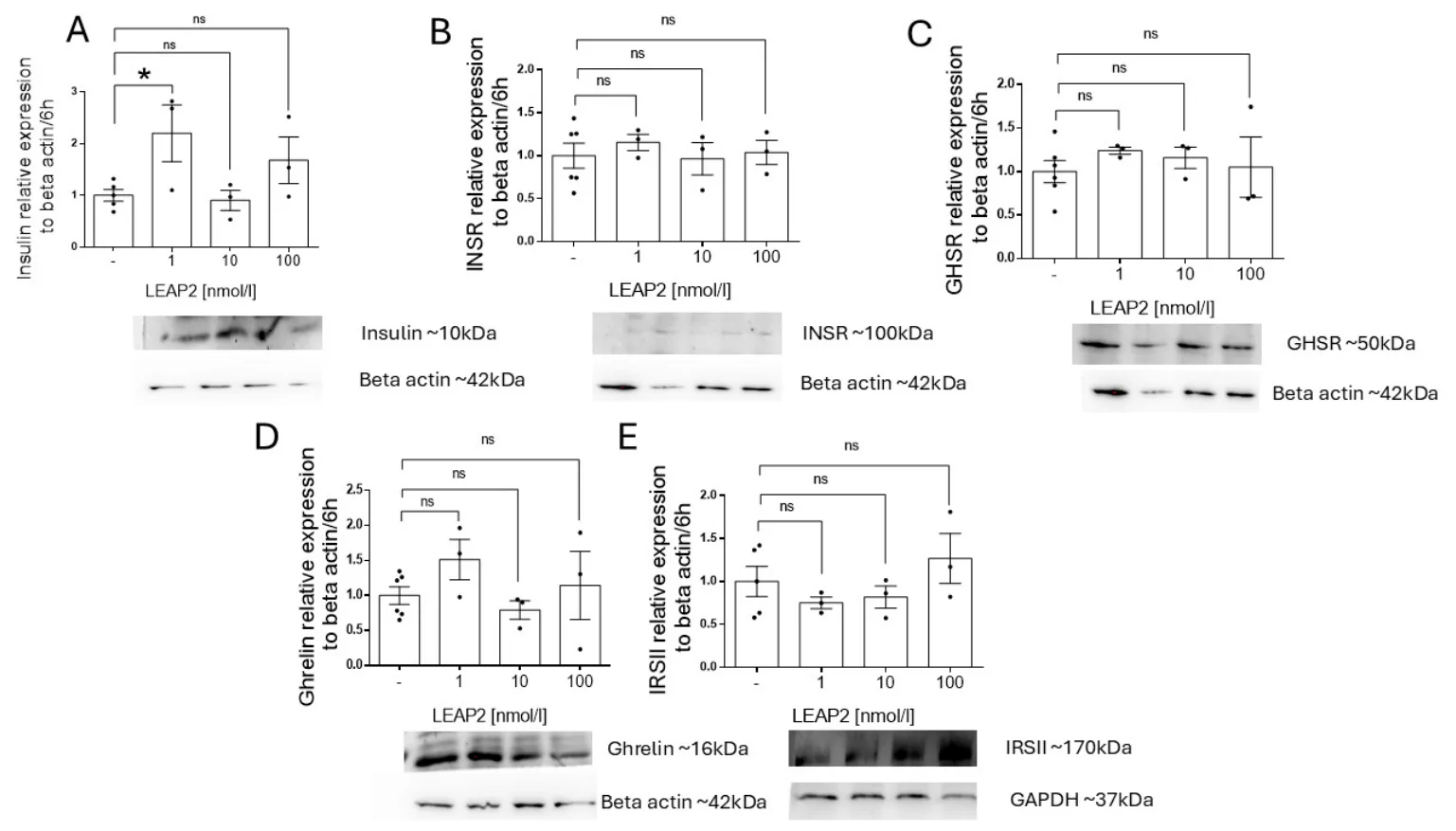
Effect of LEAP2 on the protein expression of insulin (**A**), INSR (**B**), GHSR-1a (**C**), ghrelin (**D**), and IRSII (**E**) in the INS-1E cell line after 6 h treatment with LEAP2 at concentrations of 1, 10, and 100 nM. Results are presented as mean ± SEM. Statistically significant differences between the means of the studied groups are indicated as follows: ns = not significant; * *p* < 0.05. Experiment was performed thrice using *n* = 3 per each, 3 samples were collected into one tube resulting in total of *n* = 3.

Western blot analysis for INS-1e cells exposed to LEAP2 for 6 h showed increased (*p* < 0.05) protein expression of insulin at 1 nM LEAP2 (Figure 3A). INSR, GHSR-1a, ghrelin and IRSII showed no changes (Figure 3B–E).

**Figure 4:**
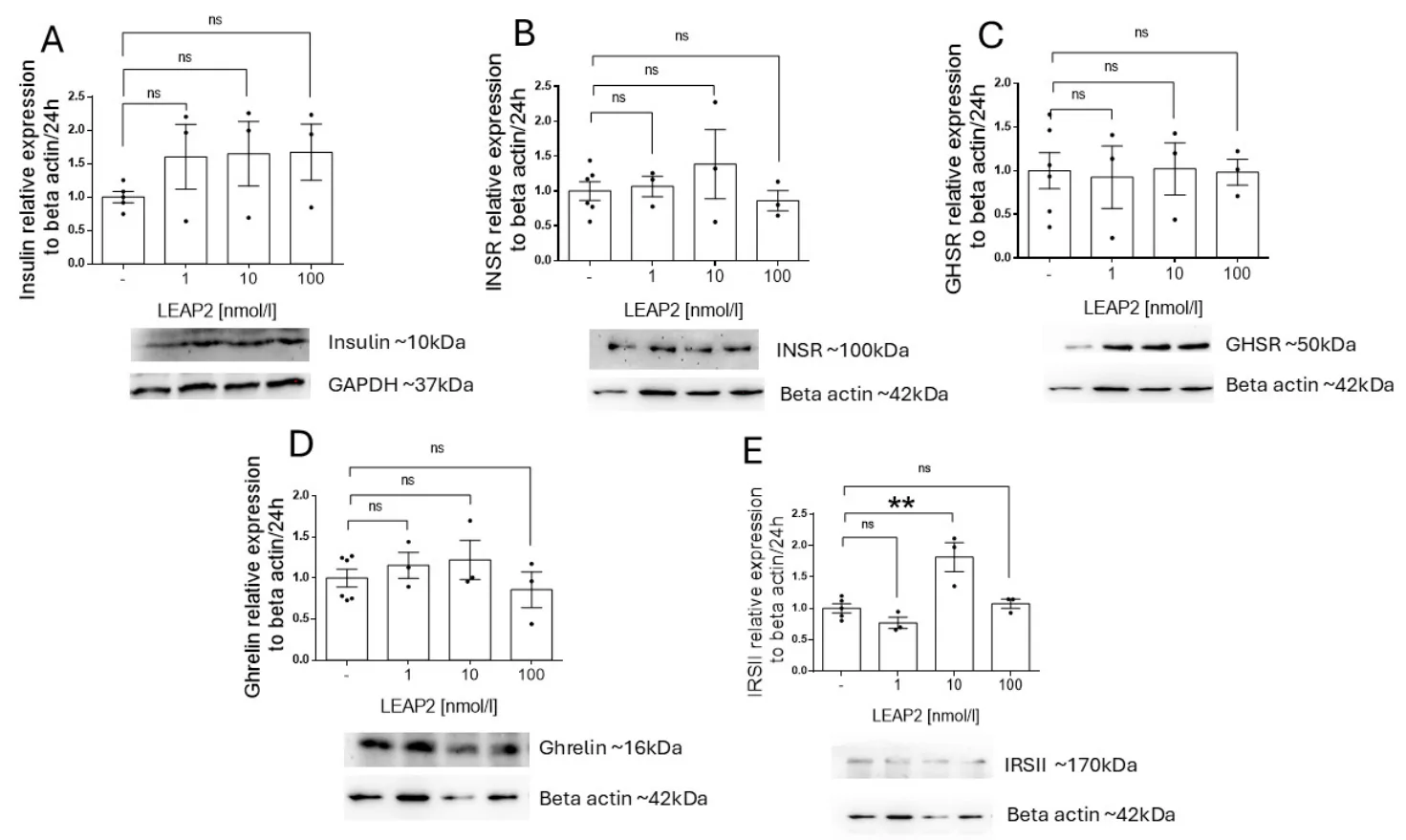
Effect of LEAP2 on the protein expression of insulin (**A**), INSR (**B**), GHSR-1a (**C**), ghrelin (**D**), and IRSII (**E**) in the INS-1E cell line after 24 h treatment with LEAP2 at concentrations of 1, 10, and 100 nM. Results are presented as mean ± SEM. Statistically significant differences between the means of the studied groups are indicated as follows: ns = not significant, ** *p* < 0.01. Experiment was performed thrice using *n* = 3 per each, 3 samples were collected into one tube resulting in total of *n* = 3.

After 24 h, IRSII showed increased protein expression (*p* < 0.01) when exposed to 10 nM LEAP2 (Figure 4D). No significant changes in protein expression were found for insulin, INSR, GHSR-1a and ghrelin. (Figure 4A–C,E).

**Figure 5:**
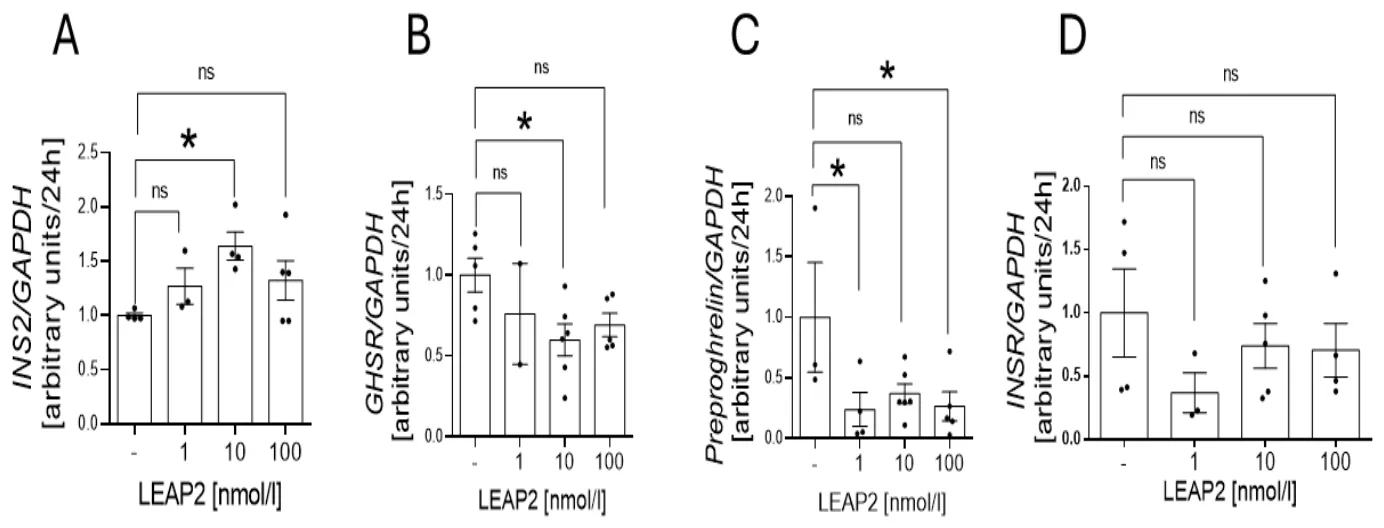
Influence of LEAP2 on the mRNA expression of INS2 (**A**), GHSR-1a (**B**), preproghrelin (**C**), and INSR (**D**) in pancreatic islets after 24 h exposure to LEAP2 at concentrations of 1, 10, and 100 nM. Results are presented as mean ± SEM. Statistically significant differences between the means of the studied groups are indicated as follows: ns = not significant; * *p* < 0.05. Experiment was performed at least twice using *n* = 2.

In case of islets, insulin 2 (Figure 5A) shows upregulated expression (*p* < 0.05) for treatment with 10 nM LEAP2, while GHSR-1a (Figure 5B) shows downregulated expression (*p* < 0.05) for 10 nM. Preproghrelin (Figure 5C) was less expressed (*p* < 0.05) at both 1 and 100 nM concentrations and INSR (Figure 5D) showed no changes.

### 2.3. LEAP2 Stimulates Insulin Secretion in β Cells and Islets

**Figure 6:**
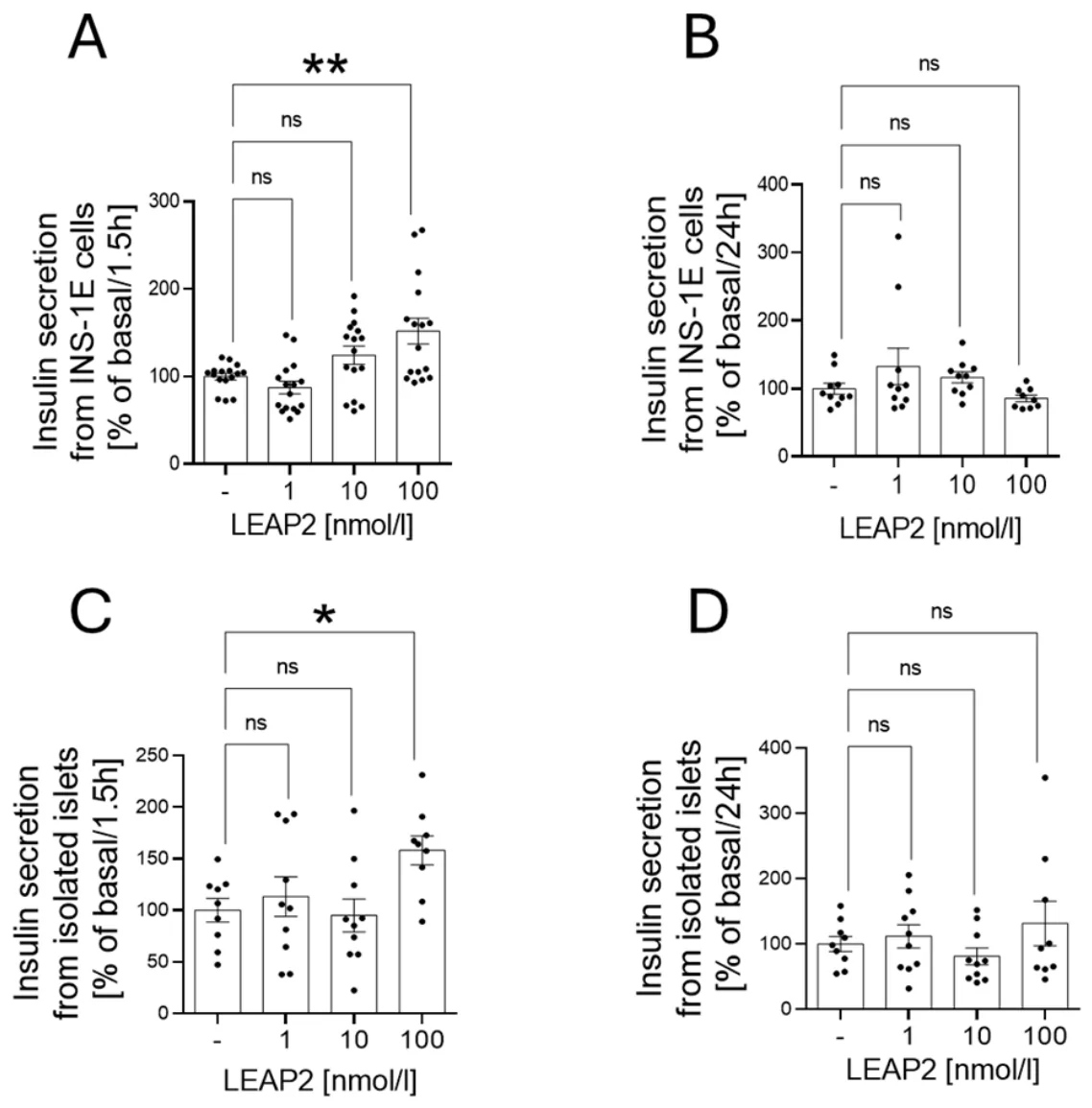
Insulin secretion by INS-1E cells (**A**,**B**) and pancreatic islets (**C**,**D**) after 1.5 h and 24 h exposure to LEAP2. Results are presented as mean ± SEM. Statistically significant differences between the means of the studied groups are indicated as follows: ns = not significant; * *p* < 0.05; ** *p* < 0.01. Experiment was performed at least twice using *n* = 5.

LEAP2 increased insulin concentration in the medium of INS-1E cells after 1.5 h exposure to 100 nM LEAP2 (*p* < 0.01; Figure 6A). A similar increase in basal insulin release was observed in isolated pancreatic islets after 1.5 h exposure to 100 nM LEAP2 (*p* < 0.05; Figure 6C). No significant effect was observed after 24 h in either model (Figure 6B,D).

### 2.4. LEAP2 Stimulates Glucose Uptake in INS-1E Cells

**Figure 7:**
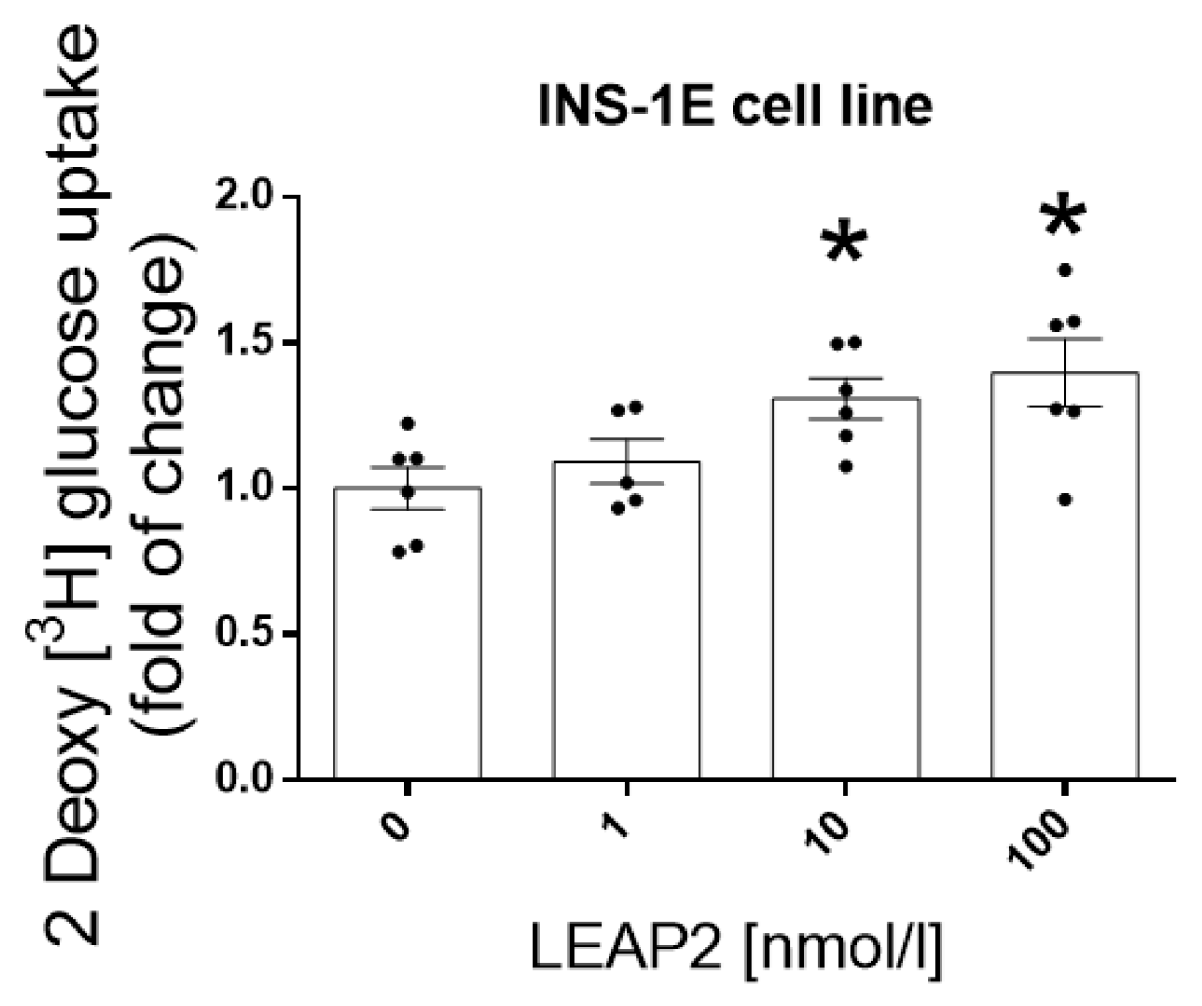
Effect of LEAP2 on glucose uptake in INS-1E cell line. Results are presented as mean ± SEM. Statistically significant differences between the means of the studied groups are indicated as: * *p* < 0.05. Experiment was performed twice using *n* = 3.

As shown on Figure 7, LEAP2 stimulates glucose uptake in INS-1E cell line at 10 nM and 100 nM concentrations (*p* < 0.05).

### 2.5. LEAP2 Has No Effect on Viability, Proliferation and Apoptosis of β Cells

**Figure 8:**
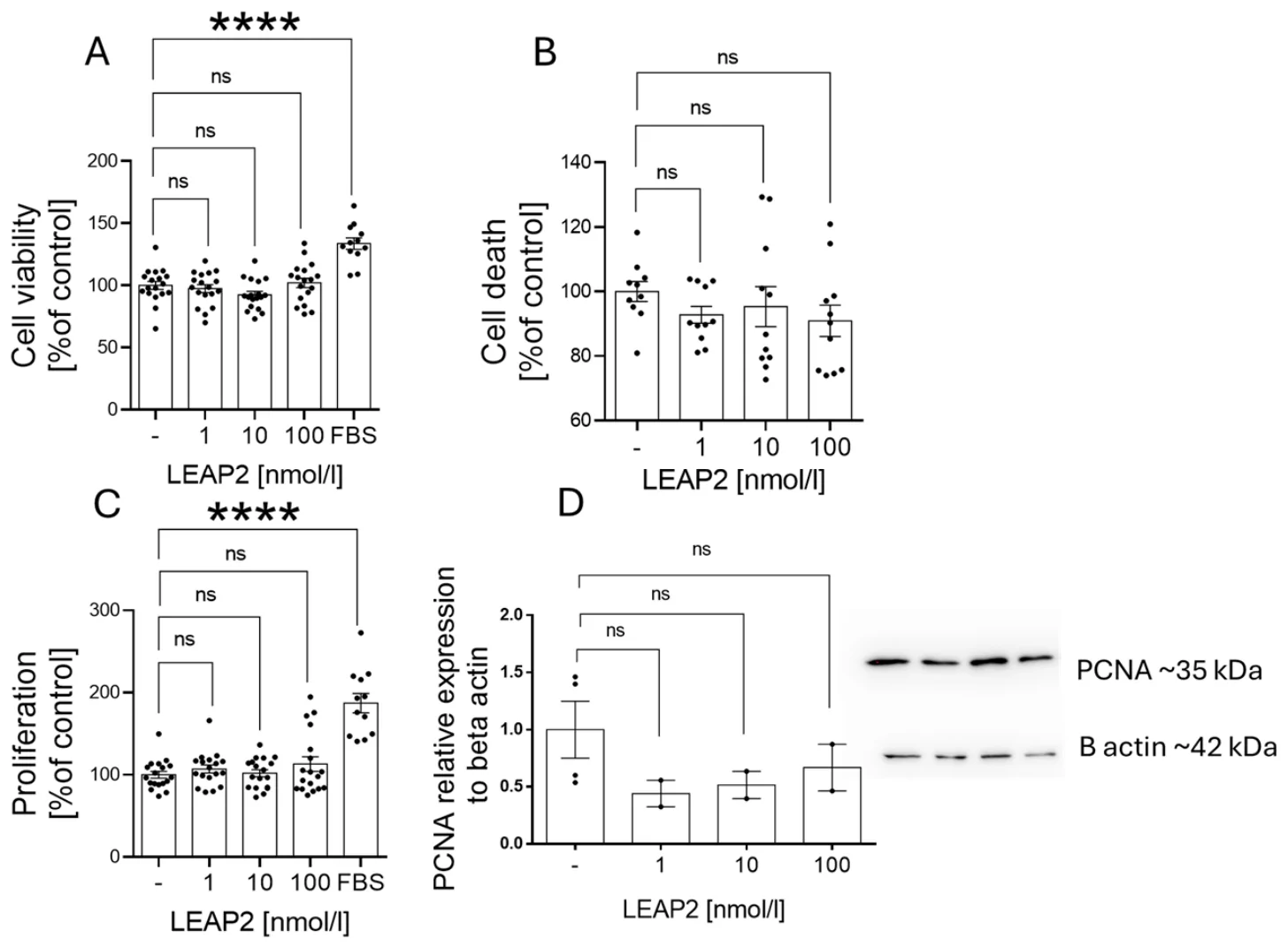
Effect of LEAP2 exposure on INS-1E cell viability (**A**), apoptosis (**B**), proliferation (**C**), and PCNA protein expression (**D**). Results are presented as mean ± SEM. Statistically significant differences between the means of the studied groups are indicated as follows: ns = not significant; **** *p* < 0.0001. Experiments were performed: twice using min. *n* = 9 for figure (**A**,**C**), twice using min. *n* = 5 for figure (**B**) and twice using *n* = 1 for figure (**D**).

MTT analysis revealed no significant changes in cell viability for INS-1e cell line after exposure to 1, 10 and 100 nM LEAP2 (Figure 8A). Cell death assay showed no changes for apoptosis under LEAP2’s exposure (Figure 8B). Proliferation also did not change, as proved by BrdU test (Figure 8C) and PCNA protein expression (Figure 8D). FBS was used as a positive control in case of cell viability as well as proliferation assays.

## 3. Discussion

In the present study we demonstrated that LEAP2 can modulate secretion and expression of insulin. We also showed effects of LEAP2 on cell metabolism, proliferation and apoptosis in β cell model. Finally, we presented the location of LEAP2 in INS-1E cells, islets and pancreas.

We chose 1, 10 and 100 nM concentrations based on physiological knowledge; 1 nM concentration is similar to physiological serum LEAP2 level in mice [2,3] and human [2,13], typically ~2 nM, while 10 nM and 100 nM were chosen as tested pharmacological concentrations [13], which showed that intravenous infusion of LEAP2 (∼25 pmol/kg/min) increased LEAP2 blood level in humans to 41.2 ± 1.1 ng/mL, about 9.32 nM. We also know that 100 nM concentration is enough to fully displace ghrelin from GHSR-1a [4,14].

In the first part of our research, PCR, Western blot and immunofluorescence staining suggested presence of LEAP2 in INS-1E cells, islets and pancreas. In those tissues, presence of GHSR-1a was also demonstrated using immunofluorescence staining. Staining of LEAP2 shows equal distribution of signal across various tissues and cells of the pancreas, without favoring any specific part. This information is consistent with reports that LEAP2 protein is primarily produced by the liver, followed by the small intestine, while other tissues such as the pancreas express a lower amount of this protein in comparison [1,3,15]. GHSR-1a immunofluorescence staining shows signal in pancreas and higher expression on outskirts of islet where α cells, δ cells and PP cells of rat are present [10]. The most likely signal comes from δ cells, as it is reported that GHSR-1a is highly expressed in this cell type compared to others [16]. Our publication shows expression of GHSR-1a in INS-1E cells, which originate from β cells, by qPCR, Western blot and immunofluorescence analysis. However, precise GHSR-1a location in islets is controversial because other works report direct ghrelin action on β cells [17] and α cells [18]. On top of that, green fluorescent protein presented location of GHSR-1a in both cell types [19]. On the other hand, ref. [16] shows no expression of GHSR-1a in β cells and a very low level of expression in α cells. Therefore, we believe that further analysis of GHSR-1a presence in specific islet cell types is necessary to clarify discrepancies.

After localization analysis, we investigated effects of LEAP2’s exposure at various concentrations on the INS-1E cell line and pancreatic islets of rat. In case of the INS-1E cell line, after 6 h expression of insulin mRNA was not significantly higher than control; however, expression on the protein level was raised. This suggests that while mRNA expression of insulin remained unchanged, translation of the protein was intensified. However, because we did not directly assess insulin translation, protein turnover, intracellular insulin trafficking or degradation, the mechanism responsible for this discrepancy remains unclear. In contrast, the increased insulin secretion observed after LEAP2 treatment could mean that LEAP2 stimulates insulin exocytosis itself, and expression is either unchanged or requires a longer incubation time period to demonstrate changes. One such possible pathway could be LEAP2-GHSR-PPARγ-Gck axis, which is reported [20] to increase intracellular content of ATP and Ca^2+^, which in turn stimulates secretion of insulin. Another explanation is increased glucose uptake caused by LEAP2, as demonstrated by us. In the first case, LEAP2 increases the amount of glucokinase and thus intensity of glycolysis and ATP production; in the second case, LEAP2 helps to deliver more glucose needed for glycolysis. Both of those mechanisms ignore expression and stimulate insulin exocytosis.

*INSR* and *IRSII* genes were chosen to research autocrine properties of insulin expression in INS-1E cells. *INSR* showed higher mRNA expression, which could hint at increased sensitivity to insulin; however, protein analysis shows no significant rise. Similar changes in protein production seem to occur after 24 h, since mRNA expression of both insulin and *IRSII* is lower while protein expression is respectively unchanged and risen. Higher protein expression of IRSII protein suggests raised sensitivity to insulin in response to LEAP2.

Experiment on islets revealed higher mRNA expression of insulin followed by lower expression of *GHSR-1a* and preproghrelin. Lower mRNA expression of preproghrelin—and its receptor *GHSR-1a*—could be the reason for raised insulin expression. Discrepancy between cell and islet models, such as lack of *GHSR-1a* and preproghrelin gene expression for cell culture, could be explained by cell heterogeneity of islets. Presence of other cell types, such as α or δ, affects insulin production [21,22,23]. Changes in GHSR-1a and preproghrelin expression could be caused by LEAP2’s influence on cell types other than β as well.

ELISA analysis revealed raised insulin secretion after 90 min exposure to LEAP2 in both β cells and islets, although there is no change for 24 h exposure period. In both models, insulin secretion was highest at a LEAP2 concentration of 100 nM. Elevated secretion from β cells was also noticed in the murine model MIN6 [20]. In this experiment, cells were cultured with LEAP2 in either 3 mM or 18 mM glucose media, and in both conditions LEAP2 enhanced insulin release. These findings indicate that LEAP2 can stimulate insulin secretion under both low and high glucose conditions, with our INS-1E results representing a response obtained at 11 mM glucose.

Our secretion results for islets are consistent with [23], where elevated insulin secretion was observed after 1 h of exposing male murine islets to 100 nM LEAP2. Rat islets exhibited a response similar to experiment on human islets [24], as the latter also secreted increased amounts of insulin after 2 h incubation with 1 µM LEAP2.

We would like to emphasize that when it comes to higher expression and secretion of insulin, shorter time is favored. Indeed, insulin showed elevated expression of protein after 6 h in β cells and elevated insulin secretion for both β cells and islets after 90 min. Secretion after 24 h showed no results, which suggests that effect of LEAP2 on secretion could fade in this period of time. This might be due to short half-life of LEAP2 in serum, which is approximately ~10–15 min in mice [25]; however, it is caused by rapid proteolytic degradation, which is not present in FBS-free medium. To summarize, based on earlier results and our research, we believe that LEAP2 causes quick but short increase in insulin secretion, as we can see a significant increase of insulin concentration in medium for β cells and islets, but only for 90 min time period. Our findings, together with those of other researchers, show that LEAP2 can enhance insulin secretion in β cells and pancreatic islets. However, an important question remains regarding how results obtained from cell cultures and animal models translate to the human organism. Notably, the first study involving LEAP2 administration in human patients [13] demonstrated an increase in postprandial insulin secretion following LEAP2 injection.

The observed increase in insulin secretion raises the question of whether LEAP2 acts directly on β-cells or indirectly through other pancreatic islet cell populations. It has been reported that LEAP2 can stimulate insulin secretion indirectly by antagonizing ghrelin signaling in pancreatic δ cells, thereby reducing somatostatin secretion [23,26]. Because somatostatin exerts an inhibitory effect on insulin release, its suppression results in enhanced insulin secretion.

Although we do believe that LEAP2 indirectly modulates insulin secretion through δ cells, our findings provide evidence for a direct effect on β cells. First, we demonstrated the expression of the LEAP2 receptor, GHSR-1a, in INS-1E cells using qPCR, Western blotting, and immunofluorescence. Second, LEAP2 treatment significantly stimulated insulin secretion from INS-1E cells. Another β cell-derived line, MIN6, also shows stimulated insulin release [20]. Finally, the increased insulin secretion observed in INS-1E cells may be explained by the results of our glucose uptake experiment. Short-term incubation with LEAP2 at concentrations of 10 and 100 nM significantly enhanced the cellular uptake of 2-deoxy-D-glucose isotope. Increased glucose uptake by pancreatic β cells is expected to elevate intracellular glucose metabolism, leading to the generation and amplification of signaling pathways that trigger insulin secretion [27]. Thus, the stimulatory effect of LEAP2 on glucose uptake may contribute to the enhanced insulin release observed in INS-1E cells. To summarize, we hypothesize that LEAP2 affects insulin secretion by both direct interaction with β cells and indirect inhibition of somatostatin secretion by δ cells.

In addition to insulin secretion, we investigated whether LEAP2 affects basic cellular functions in INS-1E cells. MTT, BrdU and cell death assays showed that LEAP2 had no effect on the INS-1E cell line’s metabolic activity, proliferation or apoptosis in any concentration. It is possible that LEAP2 has no effect on cell mitogenesis, as the HepG2 line also shows no results [28]. PCNA, a widely used cell proliferation marker, also showed no changes in expression. This could mean that LEAP2 does not affect cell mitogenesis in either an autocrine or a paracrine way, at least in the case of those two cell lines. While the INS-1E cell line is a good model to show cytotoxicity [29] and metabolic activity [30], LEAP2 does not show such an effect on β cells. Another study on the murine β cell line MIN6 shows that even higher concentrations of 1 µM LEAP2 have no cytotoxic effects on β cells [20]. However, LEAP2 shows harmful properties towards bacteria [1]. Overall, our findings suggest that LEAP2 has no effect on either β-cell metabolism or proliferation. This could be desired outcome, as there are hopes that LEAP2 could be used as therapeutic agent in future. It needs to be pointed out that our research was on the INS-1E cell line only, and we do not know the effects of LEAP2 on cell metabolism, proliferation, and apoptosis in a complete human organism; however, we know it improves the morphology of pancreatic islets in diabetic mice in vivo [20].

We are aware of limitations of our study and, although our results demonstrate a positive effect of LEAP2 on insulin production and no negative impact on cell physiology, these findings cannot be translated into medical application yet. Firstly, we did not conduct a GSIS assay, and therefore our analysis only shows a stimulatory effect on basal insulin secretion and we do not prove whether LEAP2 improves glucose-dependent insulin release. Secondly, we did not validate the specificity of LEAP2 primary antibody by peptide competition or knockout tissue staining for our immunofluorescence staining, which makes the results less credible.

Studies on cell lines and primary cultures remain preliminary as our models present several limitations. Cell cultures lack three-dimensional tissue architecture and show an inability to reproduce the complex interactions between different cell types, while islets overcome those limitations but they lack systemic regulation by other organs and vascular perfusion.

Differences between both models, especially paracrine signaling and cell heterogeneity, are also an important limitation of our study. INS-1E cells are clonal line derived from β cells and only interact in an autocrine and cell-to-cell manner. In contrast, pancreatic islets are multicellular endocrine micro-organs containing β-cells together with α-, δ- and PP cell populations. Other cell types are important for proper insulin production. Research shows that contact with non-β cells stimulates insulin production compared to single β cells [22]. Contact with α-cells stimulates insulin production as glucagon signaling activates glucagon-like peptide 1 receptor (GLP-1R) in β cells. Glucagon-like peptide 1 (GLP-1), also produced by α cells, increases cyclic AMP and production of insulin granules via GLP-1R [21]. Another example are δ cells, as they produce somatostatin which inhibits insulin production in β cells [23]. LEAP2 could have effect on those cells, which would indirectly affect insulin production, and monocellular culture would not detect it. This is especially important if we want to eventually move experimentation to the in vivo level, as potential harmful effects might not be detected by cell culture experiments. However, cell cultures can be used to prove the existence of direct interaction, as despite the lack of presence of other cell types, we report that LEAP2 stimulates insulin secretion, perhaps due to the stimulated glucose uptake presented by us.

The final limitation of our study is the fact that we used animal models, which do not always give the same results as ex vivo humans tissues and clinical trials. As of writing this paper, clinical trials on humans are rare: there are two reports of LEAP2 trials on lean and obese men [13,31]. Both of them state that LEAP2 lowers reduces food intake and limits postprandial glucose increase. Therefore, further in vivo research, especially using obese and type 2 diabetes animal models, is required to better assess the potential therapeutic properties of LEAP2.

## 4. Materials and Methods

### 4.1. Materials

Rat LEAP2 peptide was synthesized by Novazym Polska s.c. (Poznań, Poland). The INS-1E cell line was obtained from a commercially available source (Sigma-Aldrich, Darmstadt, Germany, SCC491). Fetal bovine serum (FBS) was purchased from Capricorn Scientific GmbH (Ebsdorfergrund, Germany), and RPMI 1640 medium was obtained from Corning (Corning, NY, USA). Unless otherwise specified, all other reagents used in the experiments were purchased from Sigma-Aldrich/Merck (Darmstadt, Germany). Antibodies: anti-LEAP2 (PA5-119259, Invitrogen, Waltham, MA, USA), anti–insulin (A0564, Dako, Glostrup, Denmark), anti–insulin receptor (3025, Cell Signaling Technology, Danvers, MA, USA), anti–Insulin Receptor Substrate II (sc8299, Santa Cruz Biotechnology, Dallas, TX, USA), anti–GHSR-1a (PA5-28752, Invitrogen, Waltham, MA, USA), anti–ghrelin (PA1-1070, Invitrogen, Waltham, MA, USA), anti-beta-actin (4967, Cell Signaling Technology, Danvers, MA, USA), anti-PCNA (P8825, Sigma-Aldrich, Darmstadt, Germany), anti-rabbit (7074, Cell Signaling Technology, Danvers, MA, USA), anti-mouse (7076, Cell Signaling Technology, Danvers, MA, USA), and anti-guinea pig (4031-01F, MilliporeSigma, Burlington, MA, USA).

### 4.2. Cell Culture

INS-1E cells were cultured in RPMI 1640 growth medium supplemented with 10% FBS and 1% penicillin–streptomycin and maintained at 37 °C in a humidified atmosphere with 5% CO_2_. For experimental procedures, cells were seeded into 12-well plates (for RNA extraction) and 6-well plates (for protein extraction). After 48 h of growth and attachment, the culture medium containing 10% FBS was replaced with experimental medium (serum-free medium supplemented with 0.2% fatty acid-free bovine serum albumin—BSA; Probumin^®^ Fatty Acid Free, Merck Millipore, Burlington, MA, USA) to reduce variability associated with serum components. Cells were then treated with increasing concentrations of LEAP2 (0, 1, 10, and 100 nM), for either 6 h, 12 h or 24 h, followed by RNA and protein collection for analysis.

### 4.3. Pancreatic Islet Isolation

Pancreatic islet isolation was performed according to our previously described protocol [32]. All experiments were conducted in accordance with Polish legislation governing the use of animals for scientific purposes [32]. Approval from a Local Ethics Committee for Animal Experiments was not required because the study was conducted exclusively ex vivo, no research procedures were performed on live animals, and only tissues collected after euthanasia were used. Mature male rats weighing approximately 220 g were maintained under standard institutional conditions with free access to food and water before tissue collection. After euthanasia of 3 rats by decapitation, collagenase P solution (approximately 5 U in 13 mL HBSS) was injected through the common bile duct and the pancreas was removed and digested for 20–25 min in 37 °C. Enzymatic digestion was stopped by cold HBSS supplemented with 10% FBS, followed by repeated washing. Islets were manually selected under a stereomicroscope according to size and morphology. Before experiments, islets were preincubated for at least 1 h in Krebs–Ringer buffer containing 5.5 mM glucose. Islets were used on the day of isolation.

### 4.4. Quantitative RT-PCR

Total RNA was isolated using the phenol/chloroform extraction method [33]. RNA concentration and purity were assessed spectrophotometrically, and samples with acceptable A260/A280 ratios were used for reverse transcription. Then, 1000 nanograms of RNA from each sample were reverse-transcribed using the High-Capacity cDNA Reverse Transcription Kit (Life Technologies, Grand Island, NY, USA), according to the manufacturer’s protocol. Real-time PCR was performed on the QuantStudio 12K Flex (Thermo Fisher Scientific, Waltham, MA, USA) system using 5× HOT FIREPol Eva-Green qPCR Mix Plus ROX (Solis BioDyne, Tartu, Estonia). Reactions ran for 40 cycles, 95 °C and 15 s for denaturation, 61 °C and 30 s for annealing and 72 °C and 20 s for extension. Amplification specificity was verified by melting-curve analysis. Relative expression was calculated using the 2−ΔΔCT method [34], with *GAPDH* as the reference gene, in accordance with the protocol used in our previous studies [35,36]. Primer sequences are listed in Table 1.

### 4.5. Western Blot

Cellular proteins were extracted using RIPA lysis buffer, and protein concentration was determined using the BCA Protein Assay Kit (Thermo Scientific, Waltham, MA, USA). A total of 20 micrograms of protein was separated by SDS-PAGE and transferred onto PVDF membranes. Membranes were blocked with 3% BSA in TBST for 1 h and incubated overnight with primary antibodies at the indicated dilutions. After washing, membranes were incubated with appropriate secondary antibodies, and bands were visualized by chemiluminescence. Band density was measured by densitometric analysis using TotalLab Quant v12.2 software (TotalLab, Newcastle, UK). We used peak density of band as value used in calculations. Peak density of researched protein band was divided by peak density of corresponding reference (β actin or GAPDH) band for normalization. For LEAP2 Western blotting, the expected molecular weight was considered in the low-kDa range corresponding to the processed peptide/propeptide forms; however, the presence of immunoreactive bands was not interpreted as definitive proof of antibody specificity.

### 4.6. Cell Death, Proliferation and Viability

INS-1E cells were seeded into 96-well plates and cultivated for 48 h. Following this incubation, the RPMI 1640 FBS-supplemented medium was replaced with an RPMI 1640 medium supplemented with 0.2% BSA and various concentrations of LEAP2. The experiments were conducted over a period of 24 h.

Cell death was assessed according to the instructions provided with the Cell Death Detection ELISA Plus Kit (Roche, Basel, Switzerland). Cell proliferation was measured using the protocol for the Cell Proliferation ELISA BrdU Kit (Roche, Basel, Switzerland).

Cell viability was determined by adding a solution of MTT (3-(4,5-dimethylthiazol-2-yl)-2,5-diphenyltetrazolium bromide) to the wells for approximately 20 min. Afterward, the medium was removed and 100 μL of DMSO was added to each well. The absorbance was measured using a Synergy 2 microplate reader (BioTek Instruments, Winooski, VT, USA).

### 4.7. Insulin Secretion

INS-1E cells were seeded in 96-well plates in RPMI 1640 medium supplemented with 10% FBS and antibiotics. After 48 h, the medium was replaced with serum-free RPMI 1640 containing 0.2% BSA. Cells were then incubated with LEAP2 (0, 1, 10 or 100 nM) for 1.5 h or 24 h. The culture medium was collected for insulin measurement, and MTT results from the corresponding wells were used for normalization. Insulin concentration was measured using ELISA kits from SunRed (Shanghai, China). This procedure was designed to assess LEAP2-associated changes in basal insulin release under applied culture conditions and should not be considered a standard dynamic glucose-stimulated insulin secretion (GSIS) assay.

For isolated islets, five size-matched islets were placed in each well of a 48-well plate containing Krebs–Henseleit buffer with 5.5 mM glucose and LEAP2 at 0, 1, 10 or 100 nM. Medium was collected after 1.5 h and 24 h. Normalization was based on the use of exactly five morphologically comparable islets per well, according to the protocol from previous works [36]. Insulin concentration was determined using the High Range Rat Insulin ELISA Kit cat. 10-1145-01 (Mercodia, Uppsala, Sweden) Because low- and high-glucose challenge steps were not included, the islet secretion data reflect basal insulin release rather than regulated glucose-stimulated insulin secretion.

### 4.8. Glucose Uptake

INS-1E cells were seeded in 12-well plates for 48 h. Next, cells were incubated in a glucose-free RPMI 1640 medium containing 0.2% BSA and either 1, 10 or 100 nM LEAP2 for 30 min. Next, we added a concentrated mixture of deoxy-D-glucose, 2-[1-14C] glucose (Perkin Elmer, Shelton, CT, USA) and 2-deoxyglucose to a total 18.5 kBq of deoxy-D-glucose, 2-[1-14C] glucose and 10 mmol/L 2-deoxyglucose in medium, and cells were incubated for 30 min. Then, medium was removed and ice-cold PBS containing 20 μmol/L of cytochalasin B was added to stop glucose transport. Cells were washed with PBS. The cells were lysed using 1% SDS. Lysates were transferred into scintillation liquid and β-radiation was measured using a Liquid Scintillation Analyzer Tri-Carb 4810 TR (Perkin Elmer, Shelton, CT, USA). A fraction of lysate was used for protein level measurement by BCA assay, and absorbance results were used for normalization.

### 4.9. Immunofluorescence Staining

The procedure was conducted on cells of the INS-1E line, rat pancreatic islets and rat pancreas sections. To confirm that the observed fluorescence was not due to tissue autofluorescence or nonspecific signal, a negative control was performed by incubating pancreatic slides with secondary antibodies only.

Cells were seeded on sterile slides for 48 h and fixed with 4% paraformaldehyde overnight, which was later washed twice with PBS. Cells were blocked with blocking buffer (50 mL PBS + 1% BSA + 0.01% cold fish gelatin + 50 mg of glycine) for 1 h. Then cells were incubated in blocking buffer 1:100 primary antibody solution (anti-GHSR-1a, anti-LEAP2, anti-insulin) overnight in 4 °C. Material was washed three times with 0.05% tween in PBS solution and incubated with secondary antibodies at dilution 1:1000 in blocking buffer for 2 h. Cells were washed 3 times with 0.05% tween, incubated for 3 min in DAPI (5 µg/mL in water), washed 3 times with water and prepared for observation under confocal microscope.

Pancreas sections were deparaffinized in a 60 °C dryer for 20 min and successively washed with xylene, 100%, 95% and 75% ethanol and finally with tap water. Later material was permeabilized with sodium citrate buffer (10 mM sodium citrate, 0.05% tween 20, pH 6.0) in microwave (3 × 5 min with water resupply, 20 min rest and 10 min washing in tap water), washed twice for 5 min in 0.025% Triton X-100 PBS and blocked with blocking buffer (50 mL PBS + 1% BSA + 0.01% cold fish gelatin + 50 mg of glycine). Material was incubated with anti-LEAP2, anti-GHSR-1a and anti-insulin primary antibodies at a dilution of 1:100 in blocking buffer overnight in 4 °C. Samples were washed three times with TBS supplemented with 0.025% Triton and incubated with secondary antibodies at dilution 1:1000 in blocking buffer for 2 h. After another triple washing for 5 min with 0.025% Triton, material was incubated with DAPI (5 µg/mL in water) to visualize cell nuclei and washed with 0.025% Triton once for 5 min. Results were observed with a confocal microscope.

Islets are fixed with 4% paraformaldehyde and washed with PBS twice. Then islets were permeabilizated for 1 h in 0.1% Triton X-100 solution in PBS. Islets were washed twice with PBS and blocked for 1 h with 1% BSA glycine solution (50 mL PBS + 1% BSA + 0.01% cold fish gelatin + 50 mg of glycine). Afterwards, islets were washed with PBS once and incubated with primary antibody at a dilution of 1:200 in PBS buffer with 0.2% BSA (anti-LEAP2, anti-GHSR-1a, anti-insulin) overnight in 4 °C. Next, islets were washed twice with PBS, incubated with a secondary antibody at a dilution of 1:1000 in PBS buffer for with 0.2% BSA for 2 h and washed twice with PBS. Finally, islets were incubated with DAPI at a concentration of 5 µg/mL in water for 1 min and washed twice with PBS. Results were observed with confocal microscope.

### 4.10. Statistical Analysis

Data analysis was performed using GraphPad Prism 6 software, employing a one-way ANOVA with Dunnett post hoc test compared to untreated control. Results are presented as mean ± standard error of mean (SEM). Statistical significance threshold was set at *p* < 0.05, with symbols *, **, *** and **** representing different ranges of the obtained *p*-value relative to that threshold (0.05, 0.01, 0.001, and 0.0001). Dots on columns represent individual values from the experiment.

## 5. Conclusions

The present study provides exploratory evidence that LEAP2 can modulate insulin-related responses in INS-1E cells and isolated rat pancreatic islets under in vitro conditions. The main functional observation was an acute increase in basal insulin release after 1.5 h exposure to 100 nM LEAP2 in both models. This effect was not maintained after 24 h, suggesting that LEAP2-associated modulation of insulin release may be rapid and transient under the present experimental conditions. This might be caused by the stimulation of glucose uptake caused by LEAP2, as suggested by our results on the INS-1E cell line. Importantly, these data should not be interpreted as proof that endogenous LEAP2 regulates insulin secretion in vivo.

In summary, LEAP2 affected several insulin-related functions in INS-1E cells and isolated rat pancreatic islets in vitro. Acute exposure to 100 nM LEAP2 increased basal insulin release in both models, while gene and protein expression responses were time- and model-dependent. We demonstrated evidence of presence of LEAP2 and its receptor GHSR-1a in β cells, pancreatic islets, and pancreas slides, without evidence of a distinct spatial distribution pattern for LEAP2. LEAP2 did not adversely affect INS-1E cell metabolic activity, proliferation or apoptosis. Because this study employed in vitro models, pharmacological peptide concentrations, and secretion protocols that did not directly assess classical glucose-stimulated insulin secretion, the findings should be interpreted as exploratory evidence of pancreatic responsiveness to LEAP2 rather than definitive evidence of its physiological role. Further in vivo studies are required to determine the physiological relevance of LEAP2 in pancreatic endocrine regulation.

## Figures and Tables

**Table 1:** List of primers used for quantitative PCR.

Target	Forward (5′ > 3′)	Reverse (5′ > 3′)
Insulin 1 (*INS-1*)	TCAGCAAGCAGGTCATTGTT	GGTACAGAGCCTCCACCAG
Insulin 2 (*INS-2)*	TCTTCTACACACCCATGTCCC	GGTGCAGCACTGATCCAC
Insulin receptor	CAGAAAAACCTCTTCAGGCAAT	TTCAAGGGATCTTCGCTTTC
*GHSR-1a*	CCAGAACCACAAGCAGACAG	CGAAGGACTTGGAAAAGAGGT
Preproghrelin	CCCAGAGGACAGAGGACAAG	AACATCGAAGGGAGCATTGA
*LEAP2*	GAGAACCCGGAGAATGACCC	ATCATCCCGACATGAGGCAC
Insulin receptor substrate 2 (*IRSII*)	ACCTTGCTGCTCCGTCAC	ACAGGCACTGGGAAGTTAGG
*GAPDH*	CTGCACCACCAACTGCTTAG	TGATGGCATGGACTGTGG

## Data Availability

The original data presented in the study are openly available in RepOD (Repository for Open Data) at https://doi.org/10.18150/YQILMW.
